# Cross-National Outcomes of a Digital Weight Loss Intervention in the United States, Canada, United Kingdom and Ireland, and Australia and New Zealand: A Retrospective Analysis

**DOI:** 10.3389/fpubh.2021.604937

**Published:** 2021-06-10

**Authors:** Qiuchen Yang, Ellen Siobhan Mitchell, Annabell S. Ho, Laura DeLuca, Heather Behr, Andreas Michaelides

**Affiliations:** ^1^Noom Inc., New York, NY, United States; ^2^Ferkauf Graduate School of Psychology, Yeshiva University, Bronx, NY, United States; ^3^Department of Integrative Health, Saybrook University, Pasadena, CA, United States

**Keywords:** mHealth (mobile Health), eHealth, digital health (eHealth), weight loss intervention, obesity, cross-country mHealth outcomes

## Abstract

Mobile health (mHealth) interventions are ubiquitous and effective treatment options for obesity. There is a widespread assumption that the mHealth interventions will be equally effective in other locations. In an initial test of this assumption, this retrospective study assesses weight loss and engagement with an mHealth behavior change weight loss intervention developed in the United States (US) in four English-speaking regions: the US, Australia and New Zealand (AU/NZ), Canada (CA), and the United Kingdom and Ireland (UK/IE). Data for 18,459 participants were extracted from the database of Noom's Healthy Weight Program. Self-reported weight was collected every week until program end (week 16). Engagement was measured using user-logged and automatically recorded actions. Linear mixed models were used to evaluate change in weight over time, and ANOVAs evaluated differences in engagement. In all regions, 27.2–33.2% of participants achieved at least 5% weight loss by week 16, with an average of 3–3.7% weight loss. Linear mixed models revealed similar weight outcomes in each region compared to the US, with a few differences. Engagement, however, significantly differed across regions (*P* < 0.001 on 5 of 6 factors). Depending on the level of engagement, the rate of weight loss over time differed for AU/NZ and UK/IE compared to the US. Our findings have important implications for the use and understanding of digital weight loss interventions worldwide. Future research should investigate the determinants of cross-country engagement differences and their long-term effects on intervention outcomes.

## Introduction

While initially concentrated in the United States, obesity has now also become a pervasive health concern for countries around the world (1). The prevalence of obesity since 1980 has doubled in more than 70 countries (2). In addition, countries like Australia and the United Kingdom have experienced increases at the 95th percentile in body mass index (BMI) levels, which had previously been limited to the US (3). The consequences of obesity for countries worldwide are staggering, including amplified risks of cardiovascular disease and type 2 diabetes, increased risk of mortality, and direct and indirect economic costs (2).

One of the most effective treatments for obesity is lifestyle modification (4). Through education on multiple components such as caloric intake, physical activity, and behavior change techniques, lifestyle interventions have been found to induce clinically significant weight loss (5). There are, however, significant barriers limiting the success of traditional in-person interventions. For instance, because they are time-intensive and costly, they face declining attendance rates, adherence, and ultimately decreased weight loss outcomes (6). To overcome these barriers, mobile health (mHealth) interventions are now commonly delivered via mobile platforms. mHealth interventions save time and travel costs with text-based messaging and in-app education and features. They have been found to induce significant weight loss, as well as other beneficial outcomes such as increased physical activity and improved biomarkers for risk factors of obesity (7, 8). mHealth interventions also use technology to increase adherence and improve outcomes in ways that traditional in-person care cannot, such as text message support at the time that best suits individuals (9).

This understanding, however, inherently assumes that mHealth interventions deliver equally effective care to anyone anywhere in the world. Almost all meta-analyses, systematic reviews, and commentaries about mHealth mention that a significant benefit of mHealth is its scalability, or generalizability to other populations and even other countries. Many claims are made, such as that “with little or no loss in effectiveness,” mHealth interventions developed in one place “can transcend time, culture, and language: they can be used simultaneously anywhere in the world” as is (10), or that “mobile devices are widely used across age groups and populations, providing a cost-effective platform for health program implementation. [mHealth] can be used to provide a large audience[...] with best practice approaches to treatment and prevention of obesity” (11). Yet evidence of generalizability is limited. Studies testing the effectiveness of mHealth interventions primarily include participants from only one country, most commonly the US (12). At most, studies combine many countries to constitute one category (e.g., developing countries) (13).

It is crucial to test this widespread assumption that mHealth interventions have similar outcomes in other countries, given the enormous costs of obesity for countries worldwide and the importance of providing the most effective treatments possible. Cross-country comparisons of mHealth intervention outcomes are lacking and focused on obesity prevalence rather than intervention outcomes. In addition, meta-analyses and systematic reviews make broad claims based on heavily US-centric data. For example, in one systematic review, 15 studies were conducted in the US, 2 in the UK, and one each in Australia and China (14). Thus, it is unclear whether there are underlying cross-country differences that exist, especially across different continents. Perhaps due to limited interventions that exist on a global scale, to our knowledge, analyses by country or region have rarely been conducted. To our knowledge, only two such analyses across continents exist. In one, a meta-analysis found a trend toward a difference in Internet-based weight loss intervention outcomes between continents, but performed no analyses by country, and the vast majority of data came from the United States (15). The other, a study of an mHealth physical activity intervention, found significant weight reduction in many but not all geographic regions around the world, however; countries were not analyzed separately (16). Direct cross-country comparisons are thus necessary to ascertain whether mHealth interventions truly have globally similar outcomes or whether there are consequential differences in other countries.

In particular, there is little work comparing whether an intervention primarily developed in one country (i.e., the US) can result in comparable outcomes in other similar countries around the world. Given how culturally adapted interventions can be effective (17), it is important to assess the assumption that an mHealth intervention as is, without specific cultural tailoring, can result in similar outcomes. Therefore, the objective of this study is to compare outcomes of an mHealth weight loss intervention between the country in which it was primarily developed (the United States) and three other English-speaking regions spanning three continents. To our knowledge, this is the first such cross-country comparison of the same mHealth weight loss intervention. In addition, the study retrospectively explores outcomes for better external validity, so that aspects of a formal clinical setting such as specific study timepoints or requirements do not inflate outcomes that individuals ordinarily manage on their own (18, 19). The study used a global, highly assessed mHealth intervention called Noom that has been found to result in clinically significant weight loss in healthy and at-risk populations in observational studies and randomized controlled trials (RCTs) (20–24). RCTs have found that Noom reduced weight and body fat in individuals at risk for metabolic disorders, individuals with pre-diabetes, and individuals with colorectal polyps compared to control conditions (22–24). The Noom program is based on cognitive behavioral therapy (CBT), third wave CBT such as dialectical behavior therapy (DBT), and motivational interviewing techniques, which all aid in weight loss (25–27). While the Noom program has been studied within US-based populations (20), no cross-country comparisons exist to determine its outcomes in other countries.

The study also sought to advance understanding of cross-country mHealth differences by examining engagement. Engagement is a critical determinant of intervention success. Several studies have found that increased engagement, such as logging meals or steps, contributes to increased weight loss (28, 29). Past work suggests that there can be cross-country differences in factors that impact engagement, such as usability (30). Therefore, cross-country differences in engagement could impact intervention effectiveness.

In this study, we compare outcomes over the course of the Noom intervention in four English-speaking regions, spanning a total of six individual countries. We explore how weight compares over time in each region in comparison to the US. We also examine whether differences exist across countries in engagement levels and consider how engagement is associated with weight over time. Based on previous research showing similarities in weight loss across regions (15, 16), we first hypothesize that weight loss will not be significantly different in each region compared to the US. It is unknown whether this will change over time, so we hypothesize that weight loss, as well as the relationship between weight loss and engagement, will be similar over time in each region compared to the US. Finally, based on research showing differences in factors related to engagement across countries (30), we hypothesize that levels of engagement will significantly differ across regions.

## Materials and Methods

### Regions

The US was selected as a positive control comparator to test whether the intervention is as effective in other countries. Then, to allow for a non-biased comparison (e.g., there were no translation or food content issues preventing the full intervention from being received as intended), the following countries were chosen: Canada, Australia, New Zealand, the United Kingdom (comprising Great Britain and Northern Ireland), and Ireland. These countries are similar to the US in the baseline prevalence of overweight and obese individuals (all between 60.2 and 70.1%), the main spoken language (English), and the prominence of Western foods (31). Thus, differences in outcomes could not be attributed to differing baseline prevalence in obesity or translation inaccuracies.

Similarly to past work, the following countries were combined in analyses based on geographic and cultural similarities: United Kingdom and Ireland (UK/IE), as well as Australia and New Zealand (AU/NZ). It is important to note that though these countries are geographically close, they can differ on cultural factors, climate, and ethnic makeup, among others. The regions included in this study were: the US, UK/IE, Canada, and AU/NZ.

### Intervention and Recruitment

Noom is an mHealth behavior change intervention that utilizes in-app tracking features such as food and weight logging, virtual 1:1 health coaches, behavior change techniques, and education on diet, physical activity, and psychology to enable multi-component healthy lifestyle modification. The intervention draws from CBT, third wave CBT such as DBT, and motivational interviewing techniques. These techniques are particularly used by coaches and the curriculum, which covers general psychological and behavior change principles as well as those related to diet, physical activity, and weight management. Coaches trained in CBT and motivational interviewing techniques help users to set effective goals, identify barriers, and oversee users' progress (32). Coaches interact with users via text message in the app, so users can respond whenever and for however long they prefer. Coaches check in with users at least once a week but communication can be much more or less frequent depending on user preference. Self-monitoring components (i.e., logging features) are based on empirical work showing that self-monitoring is an important behavior change technique for weight loss (33). Users are encouraged, but not required, to log their weight weekly and their food daily. Users are not required to use any certain intervention components.

Participants in all regions had access to all of these intervention components. To ensure that participants could use logging features, imperial measurements were replaced with metric measurements. Aside from this, the intervention was not culturally adapted to each country or region in order to test whether the intervention as is could effectively be applied to other countries.

The Advarra IRB approved this study. Participants for the study were selected from a pool of individuals who had signed up for Noom's Healthy Weight (HW) program based on their own motivation to lose weight. All of these participants provided informed consent that their de-identified data could be used in a longitudinal study and were given the option to opt out during the initial sign-up process for Noom. Participants were included in the study if they met the following criteria: they were between 18 and 60 years of age, began the HW program in May or June of 2019, and had at least 1 in-app action and 1 weigh-in every week over the 16 weeks of the program as a minimum threshold of activity. Data for 32,983 potentially eligible participants was pulled from Noom's database (Noom, Inc., New York, NY) and de-identified (US: 30576, CA: 822, AU/NZ: 375, GB/IE: 1,210). Participants were considered ineligible if their initial weight classified their body mass index (BMI) as underweight (<18 kg/m^2^) or healthy (18.5–24.9 kg/m^2^); if they were using the free version of the app, meaning they did not have access to the full intervention; and/or if they did not input baseline characteristics (gender and height). In addition, as in previous work, outliers were excluded, defined as an individual whose magnitude of BMI change was >3.5 within 1 month (34). A total of 18,459 participants were confirmed eligible and included in all analyses [US: 18,459 (56%); CA: 431 (52%); AU/NZ: 191 (51%); GB/IE: 597 (49%)].

### Measures

The primary outcome was self-reported weight, observed at baseline and week 16. Participants are encouraged to log their weight weekly. We also measured self-reported age, gender, and baseline BMI as predictors of weight. The country of the app store used to purchase the Noom program was used as a participant's resident country. Due to inclusion criteria for modeling purposes, there were no participants with missing data for these variables.

Engagement was measured using the total number of meals and exercises logged, messages to a coach, steps recorded, articles read, and days with at least one weight measurement each week. This includes all the fundamental components of the intervention, such as the curriculum, coaching, and self-monitoring. Each engagement variable was measured over 16 weeks. Additionally, to tap into overall engagement, a composite engagement score was calculated following previous work (20). Each engagement variable was dichotomized (0 or 1). A score of 1 was given if a participant met or exceeded the 75% percentile cut off for that engagement variable within that week. These six engagement variables were summed to calculate the overall composite engagement score (range of 0—low engagement to 6—high engagement) for each week over 16 weeks.

### Statistical Analysis

Participants' baseline characteristics were calculated using descriptive statistics, with means and standard deviations for continuous variables and frequencies and percentages for categorical variables (Table 1). Differences across regions on baseline characteristics were compared using ANOVA for continuous variables and chi-square analyses for categorical variables.

**Table 1:** Descriptive statistics for baseline characteristics by region.

**Characteristic**	**Overall (*N* = 18,459)**	**US (*N* = 17,240)**	**CA (*N* = 431)**	**AU/NZ (Total *N* = 191; AU: *N* = 149, NZ: *N* = 42)**	**UK/IE (Total *N* = 597; UK: *N* = 550, IE: *N* = 47)**	***P-*value**
**Gender**, ***n*** **(%)**					
Male	2,689 (14.5%)	2,557 (14.8%)	35 (8.1%)	15 (7.9%)	82 (13.7%)	<0.001
Female	16,770 (85.5%)	14,683 (85.2%)	396 (91.9%)	176 (92.1%)	515 (86.3%)	
Age (years), mean (SD)	44.93 (9.84)	44.99 (9.89)	44.57 (9.16)	43.2 (9.68)	44.05 (8.8)	0.008
Initial weight (kg), mean (SD)	101.51 (16.96)	101.66 (17.06)	100.06 (14.9)	98.49 (13.48)	99.16 (5.86)	<0.001
Height (inches), mean (SD)	66.02 (3.41)	66.04 (3.4)	65.35 (3.47)	65.76 (3.17)	66.17 (3.53)	<0.001
Baseline BMI (kg/m^2^), mean (SD)	30.24 (4.56)	30.27 (4.59)	30.14 (4.18)	29.46 (3.62)	29.47 (4.13)	<0.001

*ANOVAs were used for continuous variables and chi-square tests were used for categorical variables*.

To examine weight, our primary outcome and dependent variable, as a function of time, linear mixed effects models were conducted. Compared to multiple regression or ANOVA, linear mixed effects models produce more accurate parameter estimates for repeated measurements and can accommodate multiple layers of non-independence (35). Linear mixed effects models incorporating a restricted maximum likelihood approach and an unstructured covariance were used. To evaluate the impact of region, fixed effects were set as time and region, as well as their interaction, and random effects were set as time and the intercept for each participant. Time was measured as a continuous variable. Then, age, gender, baseline BMI, time, engagement, and their interactions were included in the model. The final model was determined based on chi-square goodness of fit tests. Significance tests were 2-sided except for one-sided goodness of fit tests. A standard α at 0.05 was set for significance tests. All data analysis was conducted using R (version 3.6.0).

## Results

### Baseline Characteristics by Region

Descriptive baseline characteristics, along with significance tests across regions, are displayed in Table 1. All baseline characteristics significantly differed across regions, including gender (*P* < 0.001), age (*P* = 0.008), and height (*P* < 0.001). Initial weight (in kg) significantly differed across regions (*P* < 0.001), with the highest baseline weight in the US (*M* = 101.66, SD = 17.06) and the lowest in AU/NZ (*M* = 98.49, SD = 13.48). Similarly, baseline BMI differed across regions (*P* < 0.001), ranging from a mean BMI of 30.27 (SD = 4.59) in the US and 30.14 (SD = 4.56) in Canada to 29.46 (SD = 3.62) in AU/NZ and 29.47 (SD = 4.13) in UK/IE. The final linear mixed model accounted for these differences in baseline characteristics.

### Weight Loss by Region

Descriptive statistics for weight loss over the 16 weeks are displayed in Table 2. On average, at week 16, participants in all regions had lost at least 3 kg, with up to 3.69 kg (SD = 4.35) lost in UK/IE (Table 2). In all regions, a sizable proportion of participants achieved clinically significant weight loss of 5% at 16 weeks, ranging from 27.2% in AU/NZ to 33.2% in UK/IE. At least 10% of participants achieved at least 10% weight loss at 16 weeks in all regions.

**Table 2:** Mean weight (kg) and weight change (kg) by region.

	**Overall**	**US**	**CA**	**AU/NZ**	**UK/IE**
*N*	18,459	17,240 (93.4%)	431 (2.3%)	191 (1.0%)	597 (3.2%)
Baseline	101.51 (16.96)	101.66 (17.06)	100.06 (14.9)	98.49 (13.48)	99.16 (5.86)
Week 8	98.96 (16.73)	99.11 (16.82)	97.52 (14.63)	96.18 (13.5)	96.52 (16.09)
Δ (Week 8 – Baseline)	−2.5 (2.73)	−2.49 (2.73)	−2.49 (2.80)	−2.3 (2.73)	−2.71 (2.87)
Week16	98.09 (17.02)	98.23 (17.11)	96.91 (15.19)	95.74 (13.92)	95.65 (16.24)
Δ (Week 16 – Baseline)	−3.56 (4.35)	−3.57 (4.36)	−3.26 (4.3)	−3 (4.12)	−3.69 (4.35)
Participants > 5% weight loss (%)	5,681 (30.8%)	5,482 (31.80%)	131 (30.4%)	52 (27.2%)	198 (33.2%)
Participants > 10% weight loss (%)	1,969 (10.7%)	1,831 (10.6%)	47 (10.9%)	20 (10.5%)	71 (11.9%)

*Values denoted are means, with standard deviations in parentheses except for percentages, which are denoted in frequencies and percentages in parentheses*.

In the linear mixed models, we observed significant main effects predicting weight loss as well as significant interaction effects that provide more explanation for the main effects (Table 3). For main effects, time was found to be a predictor of weight (*B* = −0.018, 95% CI: −0.19 to −0.18, and *P* < 0.001), such that weight decreased 0.02 kg per week over the 16 weeks. Characteristics such as age (*B* = −0.02, 95% CI: −0.03 to −0.01, and *P* < 0.001), baseline BMI (*B* = 3.45, 95% CI: 3.43–3.46, and *P* < 0.001), and gender (*B* = 8.82, 95% CI: 8.64–9.00, and *P* < 0.001) significantly predicted weight throughout the 16 weeks. There were no significant main effects of region for AU/NZ and UK/IE; however, participants from Canada lost a significant amount of weight compared to US participants. Across time, AU/NZ and UK/IE participants did not significantly differ in average weight compared to US participants. On average across time, US participants weighed 0.71 kg more than Canadian participants (*B* = −0.71, 95% CI: −1.15 to −0.27, and *P* = 0.002).

**Table 3:** Summary of linear mixed model results.

	**Weight (kg)**				
**Characteristic**	**Estimate**	**95% CI**	**SE**	***T*-value**	***P-*value**
Time (weeks)	−0.18	−0.19, −0.18	0.002	−76.64	<0.001
Age (years)	−0.02	−0.03, −0.01	0.003	−5.85	<0.001
Baseline BMI (kg/m^2^)	3.45	3.43, 3.46	0.007	483.29	<0.001
Engagement	0.03	0.02, 0.04	0.004	7.22	<0.001
**Gender**					
Female	–	–	–	–	–
Male	8.82	8.64, 9.00	0.09	95.65	<0.001
**Region**					
US	–	–	–	–	–
CA	−0.71	−1.15, −0.27	0.226	−3.15	0.002
AU/NZ	−0.09	−0.74, 0.57	0.335	−0.26	0.795
UK/IE	0.16	−0.22, 0.53	0.192	0.82	0.41
**Time** * **Region**					
Time * US	–	–	–	–	–
Time * CA	0.03	−0.00, 0.06	0.015	1.8	0.072
Time * AU/NZ	0.07	0.03, 0.12	0.023	3.18	0.001
Time * UK/IE	0.01	−0.02, 0.03	0.013	0.62	0.533
Time * Engagement	−0.02	−0.02, −0.01	0.0004	−33.07	<0.001
**Region** * **Engagement**					
Engagement * US	–	–	–	–	–
Engagement * CA	0.02	−0.03, 0.07	0.026	0.7	0.483
Engagement * AU/NZ	0.08	−0.01, 0.15	0.041	1.79	0.074
Engagement * UK/IE	0.04	−0.01, 0.08	0.022	1.65	0.099
**Time** * **Region** * **Engagement**					
Time * Engagement * US	–	–	–	–	–
Time * Engagement * CA	−0.002	−0.01, 0.00	0.003	−0.75	0.452
Time * Engagement * AU/NZ	0.011	−0.02, −0.00	0.004	−2.61	0.009
Time * Engagement * UK/IE	0.006	−0.01, −0.00	0.002	−2.39	0.017

Examining interactions with time allowed further exploration of weight loss over the course of the program, instead of averaged across time in main effects. Two-way interactions of time * region revealed that over time, Canadian, and UK/IE participants did not significantly differ in weight loss compared to US participants. The only significant region * time interaction was a AU/NZ * time interaction (*B* = 0.07, 95% CI: 0.03–0.12, and *P* < 0.001). Thus, US participants lost 0.07 kg more than AU/NZ participants over time, while Canadian and UK/IE participants did not differ from US participants in weight loss over time.

### Engagement by Region

Notably, participants engaged with the intervention differently across regions. One-way ANOVAs revealed that were significant differences in the average amount of engagement on five of six factors: average number of articles read, steps recorded, days with weigh ins, number of messages to the coach, and number of times they exercised each week (all *P* < 0.001, Table 4). For instance, Canadian participants messaged their coaches most (*M* = 1.84, SD = 2.58), while AU/NZ participants had the fewest messages to their coaches (*M* = 1.32, SD = 1.86), *P* < 0.001. In addition, UK/IE participants logged the most steps (*M* = 32,770.26, SD = 26,262.71), *P* < 0.001.

**Table 4:** Average weekly engagement across regions.

**Weekly engagement**	**Overall**	**US**	**CA**	**AU/NZ**	**UK/IE**	***P*-value**
Articles read, mean (SD)	14.59 (13.45)	14.58 (13.44)	15.23 (13.55)	14.59 (13.57)	14.53 (13.58)	0.001
Meals logged, mean (SD)	15.15 (6.35)	15.15 (6.35)	15.06 (6.36)	14.84 (6.4)	15.23 (6.37)	0.069
Steps, mean (SD)	30,193.83 (26,696.75)	30,058.37 (26,716.58)	31,792.84 (26,340.36)	30,808.69 (26,523.88)	32,770.26 (26,262.71)	<0.001
Days with weigh ins, mean (SD)	6.53 (1.26)	6.54 (1.25)	6.51 (1.31)	6.38 (1.46)	6.42 (1.41)	<0.001
Coach message, mean (SD)	1.55 (2.14)	1.55 (2.12)	1.84 (2.58)	1.32 (1.86)	1.5 (2.23)	<0.001
Exercise, mean (SD)	1.88 (3.11)	1.9 (3.12)	1.69 (2.97)	1.58 (2.74)	1.65 (2.99)	<0.001

In the linear mixed models, a significant engagement effect (*B* = 0.03, 95% CI: 0.02–0.04, and *P* < 0.001) was modified by the three way interaction between engagement, region, and time. Engagement appeared to matter more for US participants than UK participants (*B* = 0.006, 95% CI: −0.01 to −0.001, and *P* = 0.017) and AU/NZ participants (*B* = 0.01, 95% CI: −0.02 to −0.001, and *P* = 0.009) in predicting weight throughout the 16 weeks. With a one unit increase in engagement, US participants lost 0.01 kg more than AU/NZ participants and 0.006 kg more than UK participants over time. A two-way interaction between engagement and time was also found (*B* = −0.02, 95% CI: −0.02 to −0.01, and *P* < 0.001). Higher engagement predicted greater weight loss over time, such that one unit increase in engagement was associated with a decrease of 0.02 kg in weight over time, regardless of region of origin. No significant two-way interactions between region and engagement were found, meaning the effect of engagement on weight did not significantly differ among regions without incorporating time. These cross-region differences in engagement and weight loss are summarized in Table 5.

**Table 5:** Summary of cross-region engagement and weight loss differences.

**Region**	**Main effect (average weight loss)**	**Two-way interaction (weight loss over time)**	**Three-way interaction (engagement and weight loss over time)**
Canada	Canada > US	None	None
AU/NZ	None	US > AU/NZ	US > AU/NZ
UK/IE	None	None	US > UK/IE

*Directionality (>) denotes greater weight loss*.

## Discussion

To our knowledge, this is the first study to evaluate whether the same mobile intervention for weight loss is as effective across countries. Overall, as hypothesized, our findings showed that the Noom program generated comparable significant weight loss in the United States, the United Kingdom and Ireland, Canada, and Australia and New Zealand. Weight loss at 16 weeks was between 3% (3.0 kg) and 3.7% (3.69 kg) in all regions, matching the results of a previous study on this intervention that generated 3.5% weight loss by week 16. This is on track to surpass 5% weight loss by 1 year (20), which exceeds US Centers for Disease Control and Prevention (CDC) guidelines for significant weight loss (36). In addition, there were significant differences in engagement with the intervention across regions. Surprisingly, results also revealed that the rate of weight loss, depending on the level of engagement, differed for US participants, compared to Australia and New Zealand, and United Kingdom and Ireland. Engagement appeared to matter more for US participants in predicting weight loss than users of these regions over time. Our results extend past work on weight loss interventions, whether mobile or not, that have been focused exclusively on differences in language or culture, particularly surrounding food and physical activity (37). We found initial support for the assumption that mHealth interventions developed in one country can be applied globally to countries that share languages and attitudes toward food and physical activity. This is valuable to examine, given the ever-increasing importance of mHealth interventions for reducing obesity, widely held assumptions of mHealth generalizability, the paucity of cross-country comparisons, and the dominance of US-based results in past literature.

While no previous cross-country comparisons of an mHealth weight loss intervention existed prior, the verifiable portions of our results replicated past findings. Matching strong patterns in the literature, we found significant associations between weight and baseline BMI, gender, age, and engagement over time (20, 38, 39). In addition, we found that averaging across engagement, the intervention induced comparable weight loss over time in the US compared to UK/IE. This aligns with a meta-analysis of eHealth (Internet-based) weight loss intervention effectiveness which included a cross-continent analysis that found no differences in effectiveness between North America and Europe (13). Diverging from our results, the meta-analysis found a trend toward higher effectiveness in Australia and New Zealand compared to North America and Europe, although only 8 studies from AU/NZ and 63 from North America were included. We found greater weight loss in AU/NZ compared to the US when time was included, and no differences when time was not considered. Our results highlight the importance of conducting comparisons across countries using the same intervention and conducting mixed models rather than one-time multiple comparison tests. For instance, it could be that there were seasonality differences that would manifest over time when applying the same mHealth intervention in other locations that are not present when evaluating individual studies in meta-analyses. Future long-term studies across continents should model the effects of season. It could also be that, given the lack of a consistent pattern between main effects and interactions, we revealed a more nuanced view of weight loss between the two regions in which there was a significant difference in one but not all comparisons.

Beyond differences in demographics, there were region-level differences in how participants actively engaged with the program. For example, UK/IE participants logged the most steps and Canadian participants had the highest messages to coaches. This could be for several reasons, for example differences in sociability, time zones, and seasonality. To our knowledge, there is no prior work showing differences between the regions included in this study in terms of coach messaging. Our results with UK/IE participants and steps aligns with a study leveraging automatically recorded smartphone activity data. Results showed higher step counts in the UK than in the US (40). Future research should explore why these engagement differences occurred.

With regard to engagement cross-culturally, only one other study compared participants from Finland and India and found that while individuals had similar attitudes toward weight loss, they had very different culturally-induced engagement behaviors in a mobile physical activity app. For instance, because Indian participants viewed health as oriented around routines and not goals, they used the app's goal setting functions of the app much less than Finnish participants (41). Other previous work points indirectly to a few potential reasons for cross-region engagement differences. Past work on other mHealth interventions found differences between countries, even in similar geographic regions, on related factors that could impact engagement, such as preferences for an mHealth app's usability or attitudes toward technology, including digital literacy, data privacy, and familiarity with technology (42). Other cross-cultural research points to cultural and attitudinal differences relating to weight that can impact behavioral outcomes and therefore affect engagement behaviors (43, 44). Our results suggest that cross-region or cross-country engagement is both a more salient and complex consideration than previously thought in past studies. Notably, in our results, cross-region differences emerged across very different types of engagement factors, such as messaging a coach and weighing in. In addition, regions that had the highest engagement in one category did not necessarily have similarly high levels across other engagement variables. Clearly, cross-region or cross-country differences in engagement cannot be simply explained by static differences among countries, such as attitudes toward technology or broad cultural differences. Instead, our results highlight that there are complex factors underlying cross-region differences in engagement that are important to understand further. Future research should investigate these patterns to delineate them and why they occur.

Another reason for cross-region differences in engagement could relate to cultural tailoring. According to previous research, interventions that are specifically adapted for certain populations or locations can be effective (17). Aside from the use of metric measurements, no cultural tailoring was used in order to ascertain whether an mHealth intervention would have similar outcomes in other countries as is (i.e., without cultural tailoring). This could explain our findings of cross-region differences in engagement in that with geography-specific cultural tailoring, engagement may be similar; without cultural tailoring as in this study, cultural differences may lead to differences in engagement across countries. We examined a non-tailored intervention because of the untested assumption that mHealth interventions can be applied to other locations, even in other continents, at scale in their original form. However, future studies should assess whether culturally adapting the intervention in different countries leads to greater weight loss and fewer differences in engagement.

With the association between engagement and weight loss found in previous research and replicated in our results, it is notable that we did not just find a two-way interaction with engagement and time. Rather, we saw an additional three-way interaction with region. With high engagement, the rate of weight loss was the fastest in the US compared to AU/NZ and the UK/IE. This aligns with rare cross-cultural work, such as a study that used self-reported survey data. Results showed that US participants had greater familiarity with mHealth apps than UK participants (45). Perhaps, given their familiarity with apps, US participants were able to make best use of their high engagement with the mobile program, which translated to increased weight loss over time. However, future work is necessary to determine the precise impact of these country or region-level engagement differences on intervention effectiveness, especially over time. This study extended to 16 weeks, but the impact will likely be more consequential over a longer time period. This is an especially important question given the difficulty that interventions, whether mHealth or not, have in maintaining successful weight loss past 6 months (46).

This study had several strengths. First, in using this retrospective design, the study could explore participants' behaviors with a weight loss intervention that they were using without researcher supervision, contact, or being alerted to participation requirements along study time points. This could increase external validity of real-world conditions and contribute rare data to the literature. In addition, the study analyzed intervention effects in ways that captured the complexity of the relationships of weight loss over time, engagement, and region. Rather than merely averaging across time points or conducting one multiple comparison test in the change in weight loss at week 16, we found a more sophisticated picture of results when analyzing weight loss over time and investigating interactions with engagement. Finally, the study's extensive sample size provides greater confidence in generalizability than typical intervention studies.

The study has a few limitations. First, because the intervention was developed in the US, the sample was heavily skewed toward US participants. Seventeen thousand two hundred and forty out of 18,459 participants (93.4%) were from the US; in comparison, 431 (2.3%) were from Canada, 375 (1.0%) from Australia/NZ, and 597 (3.2%) from UK/IE. While significant interaction effects were still found with these sample sizes and they are larger than typical intervention samples, our analyses may not have captured the full range of variation in these regions. The US skew in our sample also limits generalizability of our findings. Future research should extend our findings to samples that are more equally distributed. Another limitation of the study is that no active control group was used, which means causal interpretations of the effect of Noom cannot be made, particularly in relation to the superiority of Noom compared to usual care. Future studies should use active control groups to assess the difference between the intervention and control groups across countries. Additionally, only participants in these regions who were interested in a mobile health intervention for weight loss signed up for the program and were included in the study. This same self-selection bias would apply to all four regions and the population of individuals who participate in interventions, but future studies should determine if this bias manifests differently in each of the regions. The study also used the user population of one program. While the number of participants was larger than many typical studies, the population was nevertheless naturally restricted to those who self-selected into this particular program. Future research should examine whether the results generalize to other interventions in other regions beyond these four. In addition, only one time period of 16 weeks was used. Future work should incorporate multiple time periods to explore whether cross-country effectiveness remains consistent over different periods of time and seasons. Further, the sample only included participants who met a minimum criteria of in-program activity for 16 weeks, which limits generalizability to individuals who meet this criteria. Finally, within each individual country, individuals may differ greatly on culture, primary language, country of birth, and ethnicity, which could affect engagement in ways we could not capture in this study; future studies should explore these differences and how they affect engagement.

This study provided a necessary test of a widespread implicit assumption in the literature, with important implications for how digital weight loss interventions are applied, understood, and designed worldwide. Our results provide preliminary evidence that the same mobile intervention can induce comparable weight loss in other regions or countries. In addition, even though weight loss was similar, there were country-level differences in the way people engaged with the intervention. Even with similarities of language and Western cultures, there are differences that need to be understood better in applying mHealth interventions across countries or regions. This reveals important areas of investigation for future obesity research, such as the factors determining cross-country differences in engagement, and the impact of these engagement differences over time in predicting weight.

## Data Availability Statement

Data were obtained from Noom, and legal and proprietary restrictions apply to the availability of these data. Requests to access the data should be directed to Siobhan Mitchell, siobhan@noom.com.

## Ethics Statement

The study was approved by Advarra Institutional Review Board. The participants provided informed consent to participate in this study.

## Author Contributions

QY conducted data analysis, data interpretation, and contributed to study conception and study design. EM conceived the study and contributed to study design and editing. AH performed literature searches and wrote and edited the manuscript. LD, HB, and AM contributed to study conception, study design, and editing. All authors contributed to the article and approved the submitted version.

## Conflict of Interest

QY, AH, EM, LD, HB, and AM are employees at Noom Inc.
